# KRAS Mutations Testing in Colorectal Carcinoma Patients in Italy: From Guidelines to External Quality Assessment

**DOI:** 10.1371/journal.pone.0029146

**Published:** 2011-12-21

**Authors:** Nicola Normanno, Carmine Pinto, Francesca Castiglione, Alberto Bardelli, Marcello Gambacorta, Gerardo Botti, Oscar Nappi, Salvatore Siena, Fortunato Ciardiello, GianLuigi Taddei, Antonio Marchetti

**Affiliations:** 1 Cell Biology and Biotherapy Unit, INT Fondazione “G. Pascale”, Naples, Italy; 2 Pharmacogenomic Laboratory, CROM – Centro Ricerche Oncologiche di Mercogliano, Avellino, Italy; 3 Medical Oncology, S. Orsola-Malpighi Hospital, Bologna, Italy; 4 Department of Human Pathology and Oncology, University of Florence, Florence, Italy; 5 Institute for Cancer Research and Treatment, Università di Torino, Torino, Italy; 6 Division of Pathology, Ospedale Niguarda Ca' Granda, Milan, Italy; 7 Surgical Pathology Unit, INT Fondazione “G. Pascale”, Naples, Italy; 8 Surgical Pathology and Cytopathology, Antonio Cardarelli Hospital, Naples, Italy; 9 The Falck Division of Medical Oncology, Department of Oncology, Ospedale Niguarda Ca' Granda, Milano, Italy; 10 Medical Oncology, Department of Experimental and Clinical Medicine and Surgery F. Magrassi and A. Lanzara, Second University of Naples, Naples, Italy; 11 Center of Predictive Molecular Medicine, Center of Excellence on Aging, University-Foundation, Chieti, Italy; The University of Kansas Medical Center, United States of America

## Abstract

**Background:**

Monoclonal antibodies directed against the epidermal growth factor receptor (EGFR) have been approved for the treatment of patients with metastatic colorectal carcinoma (mCRC) that do not carry KRAS mutations. Therefore, KRAS testing has become mandatory to chose the most appropriate therapy for these patients.

**Methodology/Principal Findings:**

In order to guarantee the possibility for mCRC patients to receive an high quality KRAS testing in every Italian region, the Italian Association of Medical Oncology (AIOM) and the Italian Society of Pathology and Cytopathology -Italian division of the International Academy of Pathology (SIAPEC-IAP) started a program to improve KRAS testing. AIOM and SIAPEC identified a large panel of Italian medical oncologists, pathologists and molecular biologists that outlined guidelines for KRAS testing in mCRC patients. These guidelines include specific information on the target patient population, the biological material for molecular analysis, the extraction of DNA, and the methods for the mutational analysis that are summarized in this paper. Following the publication of the guidelines, the scientific societies started an external quality assessment scheme for KRAS testing. Five CRC specimens with known KRAS mutation status were sent to the 59 centers that participated to the program. The samples were validated by three referral laboratories. The participating laboratories were allowed to use their own preferred method for DNA extraction and mutational analysis and were asked to report the results within 4 weeks. The limit to pass the quality assessment was set at 100% of true responses. In the first round, only two centers did not pass (3%). The two centers were offered to participate to a second round and both centers failed again to pass.

**Conclusions:**

The results of this first Italian quality assessment for KRAS testing suggest that KRAS mutational analysis is performed with good quality in the majority of Italian centers.

## Introduction

Mutations of the *KRAS* gene occur in approximately 40% of colorectal carcinomas (CRC), and about 90% of these mutations affect codons 12 and 13 [1]. KRAS mutations occur relatively early in colorectal tumor progression, and therefore they are usually present in the majority of the transformed cells within a KRAS mutant tumor [2]. The presence of the mutations in a restricted and well defined region of the gene and the occurrence of the mutations in an high percentage of tumor cells facilitates the detection of KRAS mutations in tumor tissues.

A number of studies have demonstrated that anti-EGFR monoclonal antibodies are active only in metastatic CRC (mCRC) patients that do not carry mutations of the *KRAS* gene. In particular, analysis of patients treated in phase II and III randomized clinical trials with anti-EGFR antibodies alone or in combination with chemotherapy, in any line of treatment, have shown that anti-EGFR agents increase the response rate and improve the progression free survival (PFS) only in mCRC patients that do not carry KRAS mutations at codons 12 and 13 [3], [4], [5], [6], [7], [8], [9]. More recently, addition of cetuximab to FOLFIRI in patients with KRAS wild-type disease was also found to result in significant improvements in overall survival (OS) [10]. Following these results, the European Medical Agency (EMEA) approved in 2009 the use of the anti-EGFR antibodies cetuximab and panitumumab only in patients with mCRC carrying a wild type *KRAS* gene. As a matter of fact, this was the first approval of a drug for a solid tumor based on a genetic test.

Following the approval of anti-EGFR antibodies for KRAS wild type CRC patients, KRAS testing has become mandatory to choose the most appropriate therapeutic strategy in mCRC. In this respect, both false-negative and false-positive results are potentially harmful for patients. In fact, false positive findings will deprive the patients of the possibility to benefit of an active treatment. On the other hand, false-negative patients might be treated with a drug that is not active. In addition, recent findings suggest that administration of an anti-EGFR monoclonal antibody in combination with a regimen containing oxaliplatin to patients with a KRAS mutant tumor might significantly reduce progression free survival [7], [9].

The introduction of a mutational assay in clinical practice has raised the issue to ensure a rapid and high quality KRAS testing to all patients. Recommendations for KRAS testing in mCRC patients were released by the European Society of Pathology (ESP) in 2008 [11]. A recent survey in 14 countries in Europe, Latin America and Asia showed that the frequency of KRAS testing in patients with mCRC increased from 3% in 2008 to 47% in 2009 and 69% in 2010 [12]. In particular, the 2010 survey revealed that test results were available within 15 days for 82%, 51% and 98% of the tested patients in the European, Latin American and Asian regions, respectively.

In Italy few surgical pathology laboratories were equipped to run molecular diagnostics at the time KRAS testing became mandatory for the prescription of anti-EGFR antibodies in mCRC patients. In order to guarantee the possibility for mCRC patients to receive an high quality KRAS testing in every Italian region, the Italian Association of Medical Oncology (AIOM) and the Italian Society of Pathology and Cytopathology -Italian division of the International Academy of Pathology (SIAPEC-IAP) started a program to improve KRAS testing. This program was based on the development of guidelines for KRAS mutational analysis in mCRC patients. Following the publication of the guidelines, the scientific societies started an external quality assessment scheme for KRAS testing, in order to evaluate the effects of guidelines on molecular diagnostic for KRAS mutations in Italy. This paper describes the development of this program that started in 2008 and was completed in 2010.

## Methods

### Methodology for guidelines

AIOM and SIAPEC-IAP identified a large panel of Italian medical oncologists, pathologists and molecular biologists that met for the first time in September 2008. Following the meeting, guidelines for KRAS testing were written by a restricted steering committee and submitted to the panel of experts for their comments. By the end of January 2009, the document was integrated with all the comments. The guidelines were published on the websites of both AIOM and SIAPEC-IAP in February 2009 (www.aiom.it, 2009; www.siapec.it, 2009). A revised version of the guidelines was prepared in 2010 and it was published in November 2010 (www.aiom.it, 2010; www.siapec.it, 2010).

### External quality assessment scheme

AIOM and SIAPEC-IAP identified a board of experts who were assigned to organize the external quality assessment scheme and that are the co-authors of this paper. Within the group three referral surgical pathology departments (Department of Human Pathology and Oncology, University of Florence; Department of Pathology, University-Foundation, Chieti; Division of Pathology, Ospedale Niguarda Ca' Granda, Milan) and three referral laboratories (Department of Pathology, University-Foundation, Chieti; Institute for Cancer Research and Treatment, University of Turin, Tuino; Pharmacogenomic Laboratory, CROM, Avellino, Italy) were identified.

Formalin-fixed paraffin embedded (FFPE) colon carcinoma specimens were collected at the referral surgical pathology departments. For each specimen, 5µ-thick slides were sent to the three referral laboratories for mutational analysis in a blind fashion. The referral laboratories analyzed the samples by using two different methods: direct sequencing of the PCR product by using in-house validated methods, and Real Time PCR with the Therascreen KRAS kit (DxS, Manchester, UK) according to manufacturer's instructions.

Slides for each center participating to the quality assessment were obtained from the selected samples. The scheme included two rounds: the laboratories that failed the first round had the chance to register for a second round.

## Results

The Italian guidelines for the mutational analysis of the KRAS gene in CRC prepared by AIOM and SIAPEC-IAP were the result of an open discussion that involved the Italian scientific CRC community. The guidelines are available at the websites of AIOM and SIAPEC-IAP (www.aiom.it; www.siapec.it) and the main points are summarized in the following paragraphs.

### Target patient population

Mutational screening of the KRAS gene should be performed in patients with mCRC for which treatment with anti-EGFR monoclonal antibodies might be indicated. Mutational analysis can be performed by using tissue from either the primary tumor or a metastatic site, since high concordance has been observed between primary tumors and metastases for KRAS mutations in the majority of the studies published up to now [13], [14], [15].

### Biological material for molecular analysis

Either frozen or FFPE tissues can be used for mutational analysis. Usually, testing for clinical purpose is performed with FFPE tissues. The primary pathologist plays a fundamental role in KRAS testing, since he has the responsibility to choose the most appropriate specimen for mutational analysis (Table 1). In particular, tissues for KRAS genotyping should contain an adequate percentage of tumor cells to avoid false negative results. However, this limit depends on the method that is used for mutational analysis [1]. International guidelines suggest that the specimen should contain at least 70% of tumor cells if a low sensitivity technique such as direct sequencing of the PCR product is used [11]. Based on the experience of our group in the last two years, we recently set this limit to 50%. In any case, the specimen should contain at least 100 tumor cells.

**Table 1:** Characteristics of the specimens for KRAS mutational analysis.

Tissues from the primary tumor or a metastatic site can be used
Testing for clinical purpose is usually performed with FFPE tissues
The specimen should contain at least 50% of tumor cells if a low sensitivity technique is used
The region with the highest percentage of tumor cells can be isolated with manual dissection
Laser microdissection should be limited to selected cases

If the tumor specimen has a lower percentage of tumor cells, the pathologist is requested to manually dissect the tissue in order to isolate the region that contains the highest percentage of tumor cells. Laser microdissection can also be used. However, this approach is not feasible as a routine clinical procedure.

### DNA extraction

DNA can be extracted from FFPE tissues by using different methods. Kits for DNA extraction from different sources including FFPE tissues are commercially available, and are usually based on the use of chromatographic columns. The use of kits has several advantages, including the shorter time necessary for the extraction and an easier standardization of the procedure. The quality and the quantity of the extracted DNA should be assessed by spectrophotometric analysis and/or agarose gel electrophoresis.

### Mutational analysis

Different methods can be used to assess the mutational status of KRAS in CRC patients (Table 2). These methods should be able to detect the seven most common mutations of the KRAS gene in codons 12 and 13: G35A (G12D), G35T (G12V), G34T (G12C), G34A (G12S), G35C (G12A), G34C (G12R), and G38A (G13D) [1]. In fact, only these KRAS mutations were investigated in the clinical trials that led to the registration of anti-EGFR monoclonal antibodies in metastatic CRC patients with wild type KRAS.

**Table 2:** Sensitivity of the main methods used for KRAS genotyping in Italy.

Method	Sensitivity*
PCR/sequencing	10–25
Pyrosequencing	5–10
PCR/RFLP	10
PCR with Stop primers and reverse dot blot	1–5
ARMS/scorpion probes (Therascreen)	1

*lower level of mutant DNA that can be detected, expressed as % of total DNA.

Direct sequencing of the PCR product represents the golden standard for mutational analysis. PCR primers should be designed to amplify codons 12 and 13 of the KRAS gene. PCR reactions should be prepared in a laminar flow hood by using gloves and filter tips, and areas for pre- and post-PCR analysis should be kept separated (Table 3).

**Table 3:** Recommendations for PCR/sequencing analysis.

PCR primers should amplify codons 12 and 13 of the KRAS gene
PCR reactions should be prepared in a laminar flow hood by using gloves and filter tips
Areas for pre- and post-PCR analysis must be kept separated
80–100 ng of genomic DNA should be amplified
Include positive and negative controls for each PCR amplification
The products of two different PCR reactions should be sequenced in forward and reverse
A mutation can be called when present in two different sequences (forward and reverse)

An adequate amount of genomic DNA (80–100 ng) should be amplified, in order to avoid artifacts that have been described to occur when a low quantity of input DNA in the PCR reaction is used. Positive and negative controls are required for each PCR amplification. We also suggest to use for sequencing at least 40–50 ng of PCR product. The products of two different PCR reactions should be sequenced in forward and reverse in order to obtain 3–4 sequences for each sample. A mutation can be called when present in at least two different sequences (forward and reverse) obtained from independent PCR amplifications.

One of the major limit of PCR/sequencing is the relatively low sensitivity (Table 2). More sensitive techniques are available to detect KRAS mutations. The Therascreen kit is a Real Time PCR-based assay the uses ARMS primers and Scorpion probes to detect the above listed seven most common mutations of KRAS in codons 12 and 13. This method is highly sensitive being able to detect KRAS mutations when they represent as low as 1% of the total DNA.

Pyrosequencing and, more recently, methods based on differential amplification of mutant DNA and hybridization with probes immobilized on a membrane are quite sensitive assays to detect KRAS mutations. However, comparative studies in large cohorts of patients with these techniques are not available yet.

The general recommendations for the preparation of PCR reactions for these latter methods are similar to PCR/sequencing. The quantity of DNA to be amplified, the conditions of amplification and the interpretation of the results are described in details by the manufacturers.

### External Quality Assessment scheme

Following the release of guidelines, AIOM and SIAPEC-IAP decided to start an external quality assessment scheme for KRAS testing in CRC that was mainly focused on genotyping. Italian laboratories that perform mutational analysis of KRAS were invited to participate to the quality assessment program. Sixty centers registered to the program at a dedicated website (www.krasquality.it). One of the centers declined, 59 centers participated to the program that started on March 15, 2009.

Three referral surgical pathologies selected 16 primary FFPE CRC with adequate content of tumor cells (>70%) (Table 4). The mutational status of the 16 samples was assessed in the three referral laboratories by using different techniques (Table 4). A good agreement on KRAS mutational status of the selected specimens was found among the three laboratories. A discordant result was found only for sample N. 6 in which PCR/sequencing detected only the G35C mutation, whereas Therascreen identified also a G34A mutant clone (Table 4). The ΔCt for the G34A mutation was significantly higher as compared with the G35C, and this might explain the lack of detection with PCR/sequencing that has a lower sensitivity as compared with Therascreen (Table 2). A meeting was held in February 2009 to select 10 samples, 5 for the first round and 5 for the second round. Samples for which a total concordance on the mutational status was found between the three referral laboratories, and from which a good yield of genomic DNA was obtained, were selected for the quality assessment scheme (Table 4). For each round 3 mutant and 2 wild type cases were chosen.

**Table 4:** Mutational status of the samples used for the quality assessment.

Sample N.	Laboratory 1PCR/Sequencing	Laboratory 2PCR/Sequencing	Laboratory 3Therascreen	Sample code*
1	G35A (G12D)	G35A (G12D)	G35A (G12D)	A1
2	G38A (G13D)	G38A (G13D)	G38A (G13D)	A2
3	Wild type	Wild type	Wild type	-
4	G35T (G12V)	G35T (G12V)	G35T (G12V)	A3
5	Wild type	Wild type	Wild type	A4
6	G35C (G12A)	G35C (G12A)	G35C (G12A)	-
			G34A (G12S)	
7	Wild type	Wild type	Wild type	-
8	Wild type	Wild type	Wild type	A5
9	G35A (G12D)	G35A (G12D)	G35A (G12D)	B1
10	G34A (G12S)	G34A (G12S)	G34A (G12S)	B2
11	G35A (G12D)	G35A (G12D)	G35A (G12D)	B3
12	Wild type	Wild type	Wild type	-
13	Wild type	Wild type	Wild type	B4
14	Wild type	Wild type	Wild type	-
15	Wild type	Wild type	Wild type	B5
16	G35A (G12D)	G35A (G12D)	G35A (G12D)	-

* = samples chosen for the external quality assessment program: A1–A5: samples for the first round; B1–B5: samples for the second round.

Five 5µ-thick slides for each sample were sent to the laboratories participating to the quality control. A random code, different for each center, was automatically assigned to the samples by an application of the website, in order to avoid exchange of information between the laboratories. The laboratories were given 4 weeks to complete the analyses and to submit the results through the KRASquality website and by fax. The laboratories were asked to submit only the results of genotyping.

**Figure 1:**
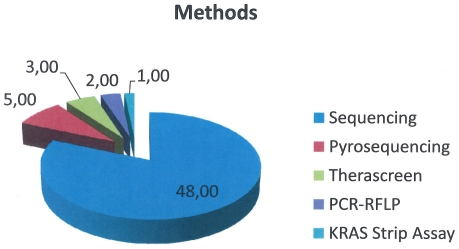
Methods used for KRAS genotyping by the centers participating to the Italian Quality assessment scheme.

All the centers submitted the results within the established deadline. Of the 59 centers, 48 (81.3%) performed the mutational analysis by using PCR sequencing, 5 (8.5%) with pyrosequencing, 3 (5.1%) with Real Time PCR (Therascreen kit), 2 (3.4%) with RFLP analysis and 1 (1.7%) with the KRAS strip assay (Figure 1).

The limit to pass the quality assessment was set at 100% of true responses. In the first round, only two centers failed to pass (3%). Both centers did not manage to extract enough genomic DNA for the mutational analyses. The samples from which the laboratories failed to extract DNA were different, suggesting that this phenomenon was not related to the quality of the specimens that they received. According to the guidelines of the scheme, the two centers were offered to participate to a second round. Again, both centers failed to pass the second round due to inability to extract genomic DNA from FFPE tissues.

The list of the centers that passed the external quality assessment scheme was published on the websites of AIOM and SIAPEC (www.aiom.it, 2009; www.siapec.it, 2009).

## Discussion

The approval of anti-EGFR agents for the treatment of mCRC patients that do not carry KRAS mutations represented a significant innovation for medical oncology. In fact, this was the first approval of an anti-tumor agent for a solid tumor based on a mutational analysis. More recently, the EGFR tyrosine kinase inhibitor gefitinib has been approved for treatment of non-small-cell lung cancer (NSCLC) patients that carry mutations of the EGFR gene. It is likely that in the next future other drugs will require the assessment of the mutational status of predictive biomarkers, represented either by the target of the drug or by signaling proteins that can affect the efficacy of the anti-tumor agent.

Different methods can be used to detect KRAS mutations in CRC specimens, and each of them has advantages and limits [1]. The approval of anti-EGFR agents for CRC without mutations of KRAS has not been linked by the regulatory agencies to a specific mutational assay. In Italy approximately 18.000 new cases of mCRC per year are expected. The Italian health system is organized on a regional basis and, with few exceptions, the majority of Italian regions did not set a limit in the number of laboratories that can run mutational analysis. Therefore, several different centers are offering KRAS testing in the different areas of the country, and a wide array of methods are employed for such analysis. For these reasons, external quality assessment is mandatory to assure that mutational testing is performed with high quality in every Italian center that provides this service.

The first external quality assessment scheme for KARS mutation testing was exclusively focused on genotyping. In fact, the aim of this first scheme was to assess the rate of false positive and negative results. The results of the scheme were exciting. The first success of the program was that the majority of the Italian centers that perform KRAS mutational analysis accepted to participate to the external quality assessment. This underlines the need of the testing laboratories to confirm the quality of their analysis through an external, independent and recognized system of evaluation. In addition, the rate of laboratories that passed was exceptionally high, since only 2 centers out of 59 failed. Although the number of samples used was limited (n.5), the threshold to pass the scheme was extremely high, since only the centers that assigned the correct genotype to all the samples passed. Nevertheless, a larger number of samples will be used in the next external quality assessment that is planned for 2012. The two laboratories that did not succeed did not manage to extract enough genomic DNA from FFPE tissues. Interestingly, both laboratories were not surgical pathologies, but genetics units that were use to run tests on blood or fresh tissues. Therefore, we hypothesize that they might have used procedures or kits for genomic DNA extraction that are not specific for FFPE specimens.

The list of the centers that passed the external quality assessment was published on the websites of AIOM and SIAPEC-IAP. The publication of this list provides to both patients and physicians the possibility to choose among a wide number of certified laboratories that are localized in different regions and that are able to provide KRAS mutational testing with adequate quality.

This first Italian KRAS quality assessment did not reveal difference in the ability to detect mutations between the techniques used by the different laboratories. However, it must be emphasized that CRC specimens with an high content of tumor cells (>70%) were selected. Different reports suggest that direct sequencing of PCR products should not be used when the specimens contains 30% or lower tumor cells [16], [17]. Although the majority of CRC specimens contains high numbers of tumor cells, a low tumor cell content might occur in mucinous tumors, or following adjuvant radio-chemotherapy for rectal tumors or in small biopsies.

The results of the Italian quality assessment scheme were superior as compared with the German program [18]. This latter external quality assessment program run 7 ring trials between 2008 and 2011, with overall 319 participants. Of these, 90.9% passed, with a failure rate of 9.1%. However, significant differences between these programs exist. In the German quality assessment, 10 samples were sent to each center and the response was due in 10 working days. In addition, 2 points were assigned for each sample (1 for the correct genotype and 1 for the specific mutation) and 1 point was deducted for a maximum of two times in case of technical failure (failure to extract or to amplify DNA). The threshold to pass the test was set at 17 points corresponding to 85% of the total score. In contrast, the threshold to pass the Italian quality control was 100% and technical failure were scored negatively at the same extent of false-negative or false-positive results.

More recently, the results of a joined regional assessment round for KRAS testing in Europe have been reported [19]. The assessment round included 59 laboratories from eight different European countries. For each country, one regional scheme organizer prepared and distributed the samples (n.10) for the participants of their own country. The samples were centrally validated by one of two reference laboratories. The results of this assessment was that only 70% of laboratories correctly identified the KRAS mutational status in all samples. Genotyping errors were made by 22% of the laboratories, whereas 8% reported technical failure. The majority of the genotyping errors were false positive or false negative results. Mistakes were made using both commercial kits and in-house validated methods.

Although direct comparison between these different quality assessment schemes cannot be drawn, we hypothesize that the educational program of AIOM and SIAPEC with the publication of guidelines followed by their presentation in a number of national meetings might have improved mutational testing in Italy. The Italian external quality assessment scheme was only related to genotyping, whereas the German and the European programs included reporting. However, in these latter schemes only genotyping was scored and, therefore, the outcomes are similar to the Italian program.

In conclusion, the results of this first Italian quality assessment for KRAS testing suggest that KRAS mutational analysis is performed with good quality in the majority of Italian centers. However, this conclusion is limited by the low number of samples employed that will be increased in the next external quality assessment, which will also include samples with low percentage of tumor cells. The Italian KRAS quality assessment scheme might represent a model for other national and international societies.

## References

[1] Normanno N, Tejpar S, Morgillo F, De Luca A, Van Cutsem E (2009). Implications for KRAS status and EGFR-targeted therapies in metastatic CRC.. Nat Rev Clin Oncol.

[2] Fearon ER, Vogelstein B (1990). A genetic model for colorectal tumorigenesis.. Cell.

[3] Amado RG, Wolf M, Peeters M, Van Cutsem E, Siena S (2008). Wild-Type KRAS Is Required for Panitumumab Efficacy in Patients With Metastatic Colorectal Cancer.. J Clin Oncol.

[4] Karapetis CS, Khambata-Ford S, Jonker DJ, O'Callaghan CJ, Tu D (2008). K-ras Mutations and Benefit from Cetuximab in Advanced Colorectal Cancer.. N Engl J Med.

[5] Van Cutsem E, Kohne C-H, Hitre E, Zaluski J, Chang Chien C-R (2009). Cetuximab and Chemotherapy as Initial Treatment for Metastatic Colorectal Cancer.. N Engl J Med.

[6] Bokemeyer C, Bondarenko I, Makhson A, Hartmann JT, Aparicio J (2009). Fluorouracil, leucovorin, and oxaliplatin with and without cetuximab in the first-line treatment of metastatic colorectal cancer.. J Clin Oncol.

[7] Douillard JY, Siena S, Cassidy J, Tabernero J, Burkes R (2010). Randomized, phase III trial of panitumumab with infusional fluorouracil, leucovorin, and oxaliplatin (FOLFOX4) versus FOLFOX4 alone as first-line treatment in patients with previously untreated metastatic colorectal cancer: the PRIME study.. J Clin Oncol.

[8] Peeters M, Price TJ, Cervantes A, Sobrero AF, Ducreux M (2010). Randomized phase III study of panitumumab with fluorouracil, leucovorin, and irinotecan (FOLFIRI) compared with FOLFIRI alone as second-line treatment in patients with metastatic colorectal cancer.. J Clin Oncol.

[9] Bokemeyer C, Bondarenko I, Hartmann JT, de Braud F, Schuch G (2011). Efficacy according to biomarker status of cetuximab plus FOLFOX-4 as first-line treatment for metastatic colorectal cancer: the OPUS study.. Ann Oncol.

[10] Van Cutsem E, Kohne CH, Lang I, Folprecht G, Nowacki MP (2011). Cetuximab plus irinotecan, fluorouracil, and leucovorin as first-line treatment for metastatic colorectal cancer: updated analysis of overall survival according to tumor KRAS and BRAF mutation status.. J Clin Oncol.

[11] van Krieken JH, Jung A, Kirchner T, Carneiro F, Seruca R (2008). KRAS mutation testing for predicting response to anti-EGFR therapy for colorectal carcinoma: proposal for an European quality assurance program.. Virchows Arch.

[12] Ciardiello F, Tejpar S, Normanno N, Mercadante D, Teague T (2011). Uptake of KRAS mutation testing in patients with metastatic colorectal cancer in Europe, Latin America and Asia.. Target Oncol.

[13] Santini D, Loupakis F, Vincenzi B, Floriani I, Stasi I (2008). High concordance of KRAS status between primary colorectal tumors and related metastatic sites: implications for clinical practice.. Oncologist.

[14] Baldus SE, Schaefer KL, Engers R, Hartleb D, Stoecklein NH (2010). Prevalence and heterogeneity of KRAS, BRAF, and PIK3CA mutations in primary colorectal adenocarcinomas and their corresponding metastases.. Clin Cancer Res.

[15] Tie J, Lipton L, Desai J, Gibbs P, Jorissen RN (2011). KRAS mutation is associated with lung metastasis in patients with curatively resected colorectal cancer.. Clin Cancer Res. 2011/01/18 ed.

[16] Carotenuto P, Roma C, Rachiglio AM, Tatangelo F, Pinto C (2010). Detection of KRAS mutations in colorectal carcinoma patients with an integrated PCR/sequencing and real-time PCR approach.. Pharmacogenomics.

[17] Tol J, Dijkstra JR, Vink-Borger ME, Nagtegaal ID, Punt CJ (2010). High sensitivity of both sequencing and real-time PCR analysis of KRAS mutations in colorectal cancer tissue.. J Cell Mol Med.

[18] Jung A, Baretton G, Dietel M, Gabbert H, Kreipe H (2009). The German quality assurance system for the molecular-pathological detection of KRAS-mutations in colorectal cancer.. J Clin Oncol.

[19] Bellon E, Ligtenberg MJ, Tejpar S, Cox K, de Hertogh G (2011). External quality assessment for KRAS testing is needed: setup of a european program and report of the first joined regional quality assessment rounds.. Oncologist.

